# Electroconvulsive therapy combined with clozapine in the management of ultra-resistant schizophrenia

**DOI:** 10.1192/j.eurpsy.2021.1387

**Published:** 2021-08-13

**Authors:** S. Khouadja, O. Charaa, R. Ben Soussia, L. Zarrouk

**Affiliations:** Psychiatry, University Hospital Of Mahdia., Mahdia, Tunisia

**Keywords:** schizophrénia, Clozpine, Electroconvulsive therapy, psychiatry

## Abstract

**Introduction:**

Although clozapine is the gold standard for treating patients with resistant schizophrenia, clinical symptoms persist in approximately 40-70% of the cases even after a year of treatment with clozapine. Electroconvulsive therapy (ECT) has been tried as augmentation therapy in the management of ultra-resistant schizophrenia.

**Objectives:**

To review recent studies concerning the effectiveness of ECT associated with clozapine in the management of ultra-resistant schizophrenia.

**Methods:**

This is a review of the literature via Medline and Sciences direct. The database was searched using the keyword combination “clozapine” with “ECT”, “resistant schizophrenia” with “ECT and clozapine” and “clozapine resistant schizophrenia” with “ECT” from 2010 to 2020.

**Results:**

We found 4 reviews and meta-analyzes and 6 studies. According to the majority of recent reviews and meta-analyses studied, patients who were resistant to clozapine responded to the combination of clozapine and ECT in 54% of the cases. ECT by increasing the permeability of the blood-brain barrier facilitates the brain transmission of large molecules such as clozapine, thus promoting better efficacy of clozapine. The combination of ECT with clozapine was generally well tolerated in the majority of patients. The most frequently reported adverse reactions in the literature were memory impairment and headache. These effects did not appear to be chronic or persistent, but rather transient and mild. Other rare cases such as prolonged seizures, tachycardia, and confusion have been reported.

**Conclusions:**

ECT associated with clozapine is an effective, relatively safe and tolerable treatment in the majority of cases.

